# New cases of HIV/AIDS in adults during 2007-2012

**DOI:** 10.1186/1471-2334-13-S1-P6

**Published:** 2013-12-16

**Authors:** Andreea Cristina Stoian, Constantin Bănică, Loredana Ionescu, Augustin Cupşa, Lucian Giubelan, Florentina Dumitrescu

**Affiliations:** 1 University of Medicine and Pharmacy Craiova, Romania; 2 “Victor Babeş” Clinical Hospital of Infectious Diseases and Pneumology, Craiova, Romania

## Background

We analyzed the epidemiological and clinical aspects in patients newly diagnosed with HIV.

## Methods

We performed a retrospective, compared study, in the Regional Monitoring and Evaluation of HIV/AIDS from Craiova, between 01 January 2007 – 31 December 2012, in two groups of patients with HIV/AIDS, as follows: group A-59 patients diagnosed with HIV/AIDS in the period 01 January 2010 – 31 December 2012 and group B-113 patients diagnosed with HIV/AIDS in the period 01 January 2007 – 31 December 2009, the results analyzed were: clinical, epidemiological, immunological and virological evolution in the first 12 months of screening. For statistical analysis we used the Epi Info program, threshold (p) values are statistically significant at ≤0.05.

## Results

Demographic data for group A vs. group B: female/male = 16 (27.11%) / 43 (72.88%) vs. 61 (53.98%) / 52 (46.02%); p=0.0011, urban/rural = 30 (50.84%) / 29 (49.15%) vs. = 65 (57.52%) / 48 (42.47%), p=0.42; probable route of transmission: sexual/horizontal, in early childhood / i.v. drugs = 49 (83.05%) / 9 (15.24%) / 1 (1.69%) vs. 62 (50.44%) / 51 (45.13%) p=0.002; the average age (years): 31.2±10.98 vs. 24.5±12.25; p=0.0005; the average CD4 count = 323.14±161.57 vs. 209.22±104.61 cells/cmm; p=0.86, the average HIV viral load = 5.4 log copies/mL vs. 4.9 log copies/mL, clinical and immunological distribution: B3 = 11 (18.64%) vs. 18 (15.92%), C3 = 15 (25.42%) vs. 43 (38.05%), the symptomatic/asymptomatic onset = 45 (76.27%) / 14 (23.72%) vs. 80 (70.79%) / 33 (29.20%); p=0.47, the most common opportunistic infection is pulmonary tuberculosis = 19 (32.20%) vs. 31 (27.43%); p=0.59; administration of ART: 45 (76.27%) vs. 80 (70.79%); deaths in the first 12 months of detection = 2 (3.38%) vs. 3 (2.65%); p =1.000.

## Conclusion

The new cases of HIV infection in 2007-2009 were lower than during 2010-2012, when the route of sexual transmission was predominant, male sex is more commonly affected, and the average age is elder. Mortality remained at a low rate, but similar in both periods followed (2007-2009 vs. 2010-2012). Tuberculosis was the most common opportunistic infection in HIV detection, independently of the period followed.

